# Misconceptions about trigger finger: a scoping review. Definition, pathophysiology, site of lesion, etiology. Trigger finger solving a maze

**DOI:** 10.1186/s42358-024-00379-7

**Published:** 2024-07-11

**Authors:** Eslam Shohda, Reda Ali Sheta

**Affiliations:** 1 Al-Ahrar Teaching Hospital, General Organization For Teaching Hospitals and Institutes, 5Th Kamal eldeen Abaza Street from, Manshet Abaza, Zagazig, 44759 Al-Sharkia Egypt; 2 Al-Ahrar Teaching Hospital, General Organization For Teaching Hospitals and Institutes, 1st Talaat Harb Street from El Salm Street. Beside Sednawey Hospital, Zagazig, 44759 Al-Sharkia Egypt

**Keywords:** Trigger finger, Injection; pathophysiology, Surgical management, Histology

## Abstract

Trigger finger (TF) is a disorder characterized by snapping or locking a finger. It has a prevalence of greater than 3% in the general population; however, this estimate could be increased to 5% up to 20% in diabetic patients. Some unreal ambiguity about definition, pathophysiology, site of lesion, and etiology are found among researchers and clinicians, leading to a lack of understanding of all aspects of the disease and improper management as many clinicians proceed to anti-inflammatory medications or steroids injection without in-depth patient evaluation. Original articles cited up to 2022, found through a Google search using the specified keywords, have been used in this review. Close-access articles were accessed through our researcher account with the Egyptian Knowledge Bank. In this review, we will focus on pathophysiology to present all possible findings and etiology to represent all risk factors and associated diseases to assess and confirm a diagnosis and the exact location of pathology hence better treatment modalities and reducing the recurrence of the pathology.

## Introduction

**Fig. 1:**
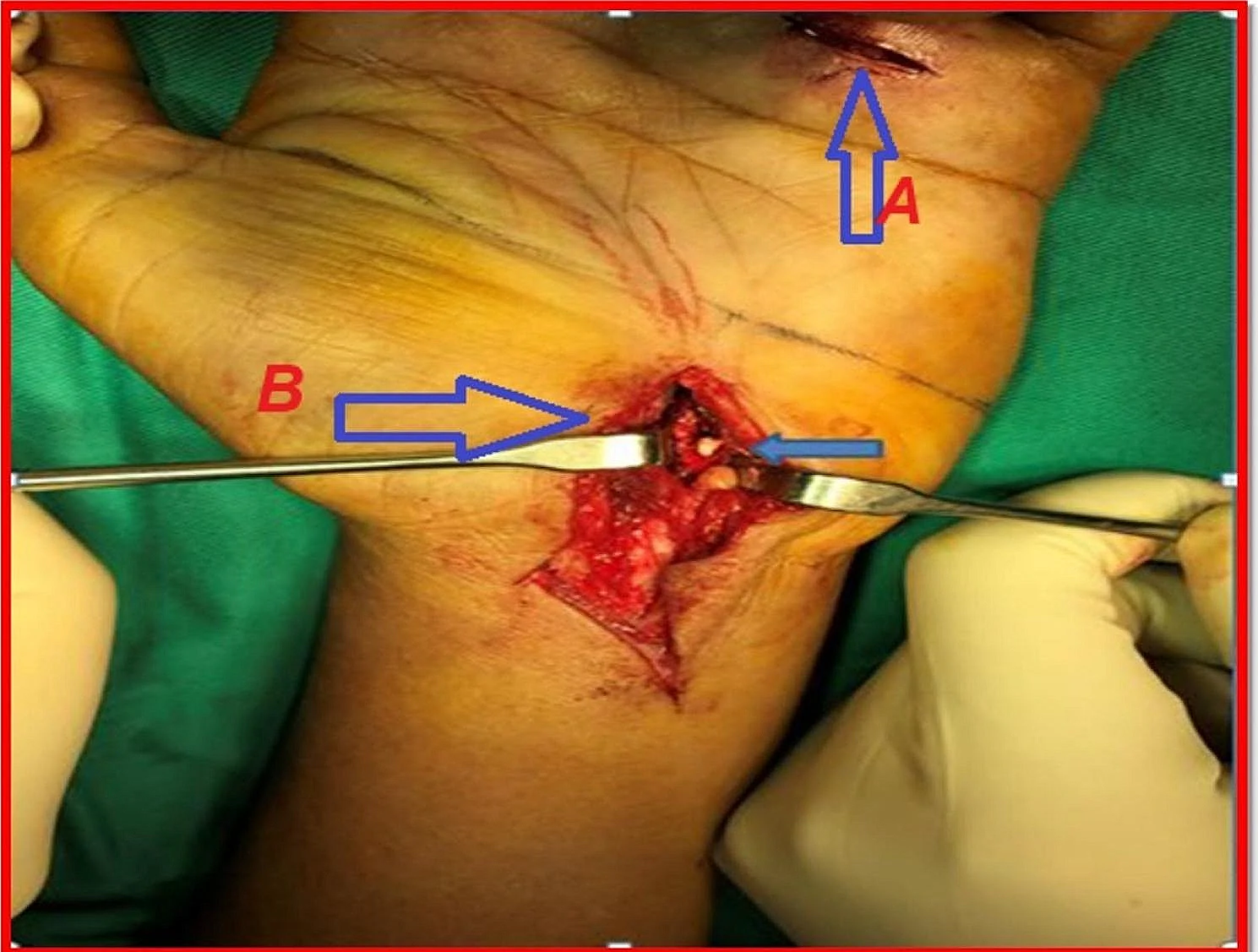
Initial A1 pulley release of the left ring finger Intraoperatively (**A**) but finger still remained in flex position. Chalky white material was found in The flexor digitorum superficialis (FDS), tendon sheath proximal to the flexor retinaculum (**B**) [28]

A trigger finger is painful popping or clicking sound to be elicited by flexion and extension of the involved digit. It is a multifactorial disease [1]. Some researchers define TF as stenosing tenosynovitis, but the literature doesn’t support the mandatory inflammatory pathology or the mandatory tendon or tendon sheath affection. Although A1 pulley is the most common site of pathology, other sites can be the site of the pathology, and even in some cases, the pulley is not affected. Still, the tendon is involved [2], so the clinician should examine all possible affection sites (Fig. 1).

## Pathophysiology

**Fig. 2:**
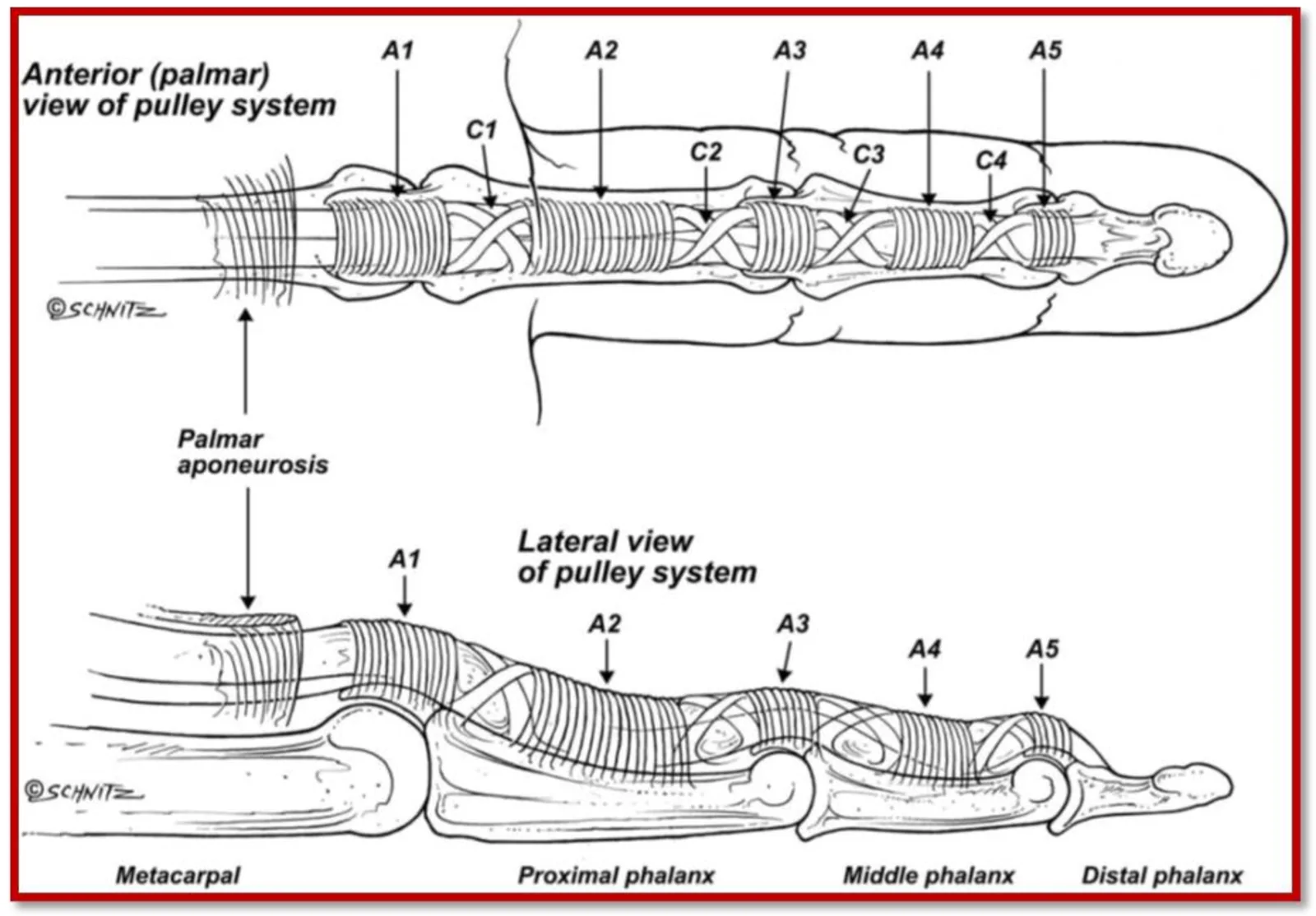
The digital pulley system of the fingers [58]

**Fig. 3:**
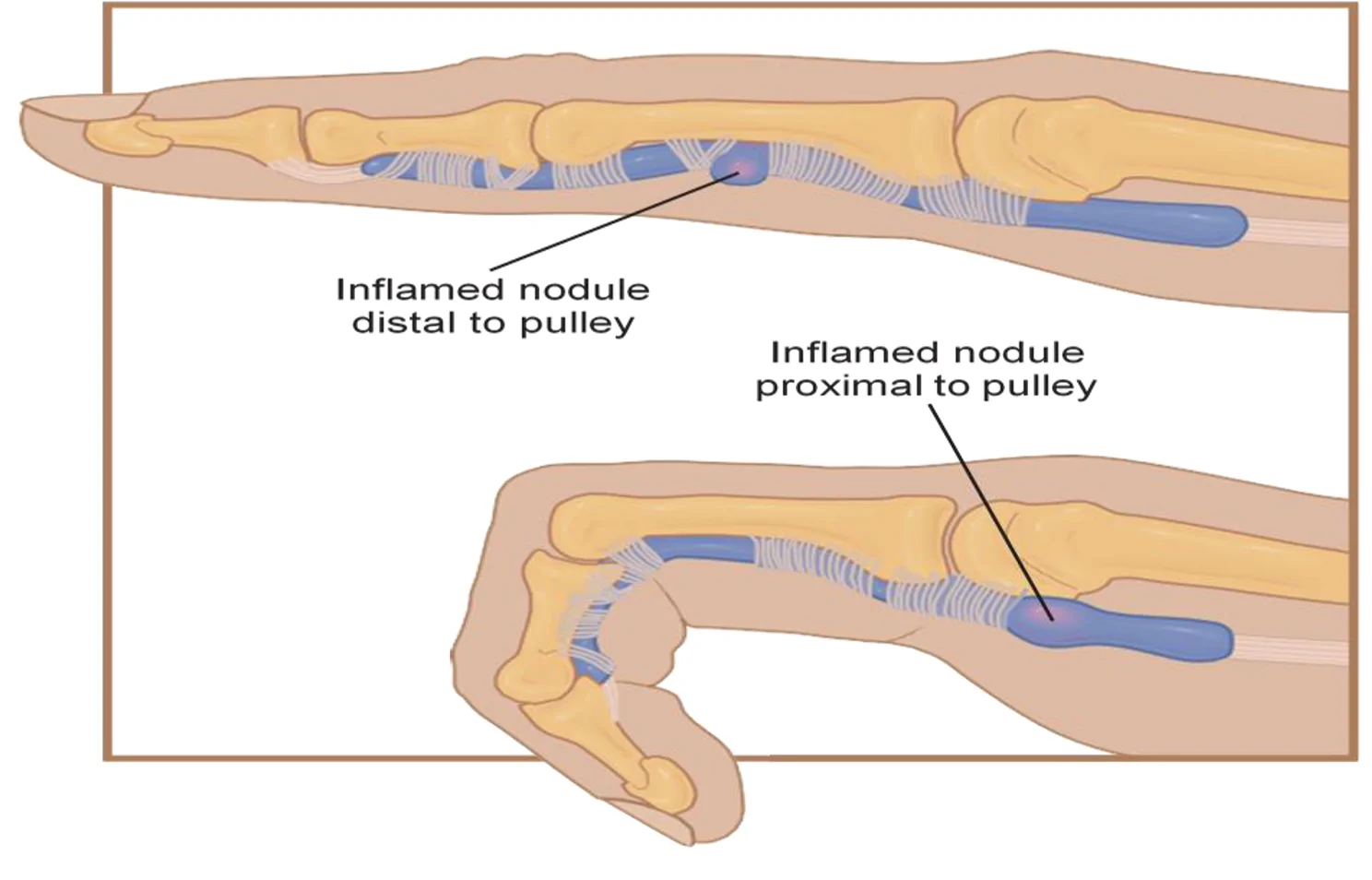
The mismatch of diameter between flexors tendons and the annular pulley [59]

The common mechanism of the disease is a mismatch of diameter between the flexor’s tendons and the annular pulley of the finger mainly A1 (Fig. 2). However to some extent, triggering occurs at A2 or A3 pulleys [3, 4, and 5] (Fig. 3).

**Fig. 4:**
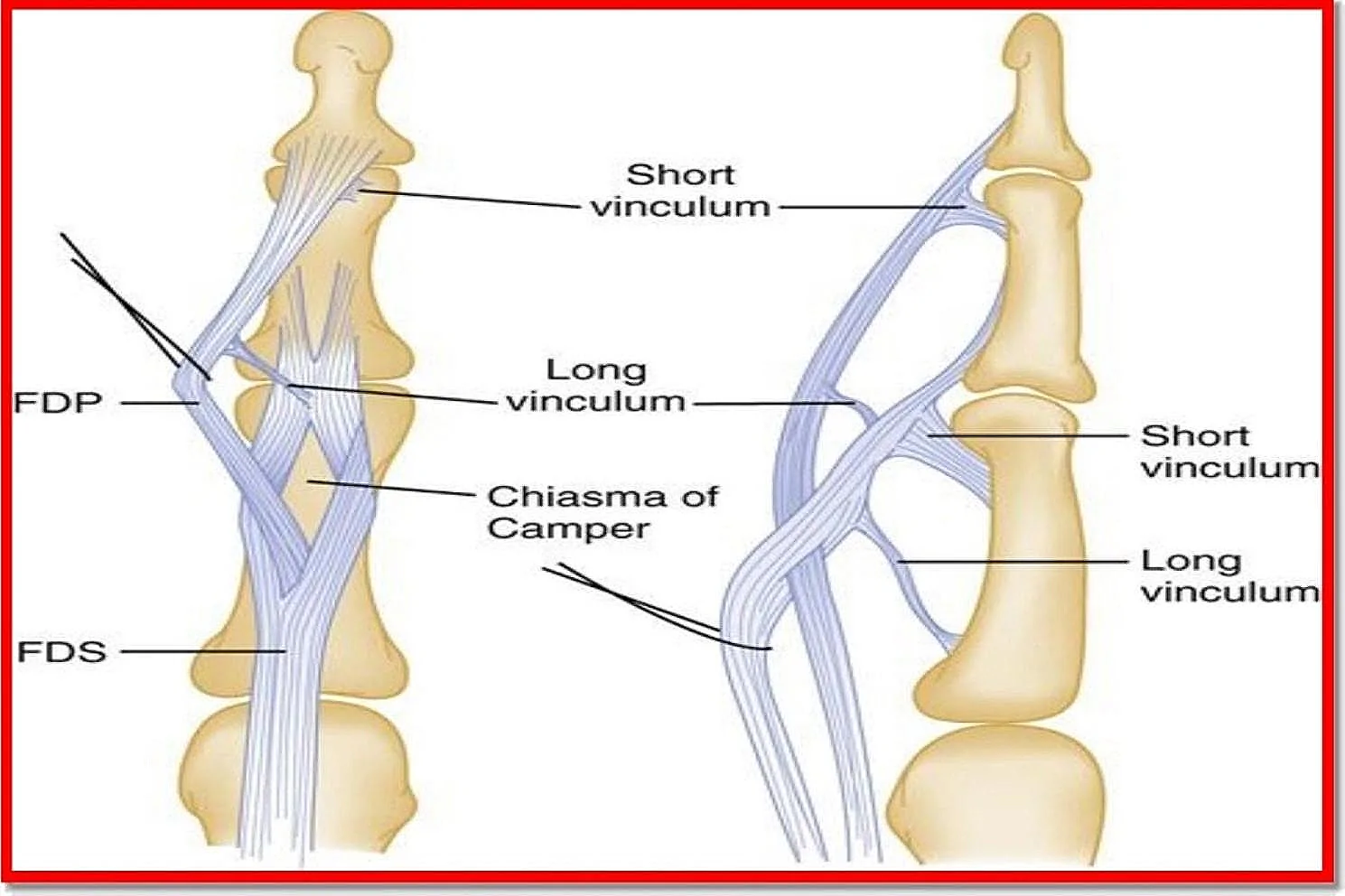
Camper’s Chiasm (decussation of the flexor digitorum superficialis) [59]

The second site for triggering is at the decussation of the flexor digitorum superficialis (Camper’s Chiasm) (Fig. 4); it occurs along flexor tendons at their entrance to the fibrous sheath. One case report found infiltration with amyloid particles at this site [6].

The third trigger site is at the palmar aponeurosis (A0 pulley or PA pulley), which acts as a flexor tendon. Wu et al., 2021 found that PA pulley was the primary cause of 31–47% of trigger fingers [7].

The fourth site for triggering is the flexor retinaculum at the wrist, which in these cases acts as a pulley for the passage of flexor tendons. The pathology was a mass within some flexor tendons (e.g., tophaceous gout, hemangioma, ganglion cyst, schwannoma, fibrous histiocytoma) [8].

The fifth site for triggering is not at the palmar surface of the hand as usual, but it is done at the dorsal surface of the hand due to impingement of one or more extensor tendons on the extensor retinaculum at the wrist. Tendon fraying, anomalous muscle, and the dorsal extensor retinaculum thickening are possible pathological findings [9].

The sixth site for triggering occurs at the side of the finger by disturbance of the volar gliding of the lateral bands of the extensor apparatus on the lateral aspects of the proximal interphalangeal joint. This occurs in rheumatoid patients due to synovial involvement, swelling, capsular thickening, and bony destruction. It occurs when the metacarpophalangeal joint is extended, and the patient actively flexes the proximal interphalangeal joint [10].

It is thought that Notta,1850 was the first physician who investigated TF. He noticed a node (Notta node) above the inferior palmar crease (A1 pulley). Since that time, physicians and researchers have focused on the A1 pulley as the site of the problem. Barnard (1903) resected the A1 pulley in TF patients, and this procedure solved the problem, so most of the attention was directed toward A1. Hence, he thought that the origin of this node was the flexor tendon or the synovial fluid cavity (annular pulley) [11].

According to some TF ultrasonography assessment studies, it was found that some possible changes in pulleys are: thickening of the pulley which may be global or nodular **(so, presence of Nota node is not inevitable)**, pulley hypervascularization, and cyst. Potential changes in flexor tendons are loss of regular fibrillar echogenic pattern, irregularity or blurring of the tendon margin, and fluid collection (cyst) in the tendon sheath (tendinosis or tendinopathy). Still, flexors tendon changes are not consistent findings [12]. Although studies found that pulley changes are 100% of cases studied, Keem and Lee, 2010 found that 30% of clinical trigger finger cases studied showed abnormality of tendon and its sheath without any abnormality of A1 pulley [2], and this may explain that pathological changes are according to underlying causes and site of triggering.

Some histopathological changes to the affected pulley are connective tissue irregularity of the inner layer of the A1 pulley. The diameter of the collagen fibers is smaller than in controls (due to an increase of collagen Type III). There is an increase in the extracellular matrix and a characteristic increase of chondroid-metaplasia compared with controls [13]. The underlying tendon histologic features are characterized by separated, disorganized, and disrupted collagen fibers, areas of hypercellularity and hypocellularity, and an increased amount of ground substance both around collagen fibers and between bundles. Occasionally, flexor tendinosis may be complicated with tendon tears, intratendinous ganglion cyst formation, or longitudinal tendon splits [14]. The underlying flexor tendons often become swollen and, on a transverse scan, their cross-sectional area is rounder than that of the adjacent unaffected tendons. In the acute stages, synovial sheath effusion may develop and is more evident proximal to the thickened pulley [14]. While some studies found inflammatory cells as a part of histopathology, others didn’t find them, so we can’t say TF is a stenosing tenosynovitis in all cases of TF. Other pathological findings will be explained while discussing the etiology.

## Etiology

Most researchers and clinicians deal with TF as an idiopathic disease. However, the extensive review reveals that TF is a multifactorial disease. It was found that treating associating diseases is basic in treating TF [15]. There is enough evidence associating TF with some specific diseases. Diabetes mellitus (DM) is the most common disease associated with TF with prevalence in diabetics is ranged from 1.5% to 20, Löfgren et al. found that DM is an important risk factor for developing TF even with adjusting for sex, age, BMI, manual work, statin use, smoking, and alcohol consumption [16]. Several mechanisms could explain the increased risk of developing TF in diabetic patients as Tendinopathy which is a common complication in diabetics. The imaging and histological studies have revealed pathological changes in various tendons of patients with diabetes, including disorganized arrangement of collagen fibers, microtears, calcium nodules, and advanced glycation end product (AGE) deposition. Shi et al. 2021 AGEs which are the result of hyperglycemia, create pathological collagen cross-links and are accumulated in tissues such as tendons resulting in a thicker, stiffer, and tougher tendon [17].

Kameyama et al. found that granulation tissues and myxomatous degeneration are found in diabetic TF more than nondiabetics [18]. Cain et al. found that mRNA expression of COL-I, COL-II, and aggrecan was significantly higher in the pulley A1 of diabetic patients as compared to no diabetic TF patients, this account for disorganized collagen arrangement and thickened pulley (glycosylation of collagen) [19]. Some studies have found that the risk of trigger finger in diabetes is associated with female gender, age over 60 years, long duration of diabetes, insulin dependency, high HbA1c, neuropathy, nephropathy, and retinopathy [16, 20].

There is a reported association between carpal tunnel syndrome (CTS) and TF. It was found that concomitant CTS is a risk factor for both initial and subsequent TF. Ferree et al., 2012 found that about 22% of studied TF patients had a recurrent triggering, but on the other fingers, at follow up and found that CTS or type 1 diabetes are risk factors for that second TF [21]. Kumar and Chakrabati, 2009 found that 43% of studied TF patients also had CTS [22]. In a prospective study by Chammas et al., CTS was found to be six times more frequent in patients with type I diabetes and four times more frequent in patients with type II diabetes [23]. It is thought that entrapment and compression are due to connective tissue proliferation, which is common in diabetics; however other space-occupying lesions in the carpal tunnel may cause both CTS and TF, whether they have diabetes or not.

The reported incidence of trigger digits after carpal tunnel release (CTR) is ranged from 5.2 to 31.7%. The time to development of trigger digits was approximately six months postoperatively [24]. Postoperative edema, inflammatory reaction, and scar tissue are possible explanations for triggering. In a cadaveric study, Karalezli et al.,2013 found that CTR increases the entrance angle of flexor tendons to A1 pulleys which may increase tension at the pulley, and this is another explanation of triggering after CTR [25]. Extra-articular deposition due to gout, pseudo gout, or Amyloidosis can lead to TF. Many case reports revealed that monosodium urates (gout) precipitated through various hand structures leading to TF. Tophaceous gouty nodules may be precipitated within the synovium and paratenon of the flexor superficialis (sublimes) and profundus tendons. Nodules may be precipitated within the substance of the tendons and \or the epineurium on the median nerve (so carpal tunnel syndrome may be accompanied by TF). Flexor tendons may be hypertrophied and encased with gouty deposits [26].

According to reported cases, various sites may be affected separately or with each other [26]. Inflammatory substances were found to accompany gouty TF [27]. In one reported case, the patient denied any prior episodes of gouty arthritis, so gouty TF may be the first manifestation of gout [28].

Calcium pyrophosphate crystals may be precipitated within any flexor tendon and mixed with inflammatory cells. The entire flexor tendon sheath may be edematous and contain a significant amount of serous fluid [26].

Amyloidosis is a disorder in which insoluble amyloid proteins are deposited in some body organs, causing abnormal protein build-up in tissues and leading to organ dysfunction and even death. There are some types of Amyloidosis according to a type of protein [29].

Asencio et al. 1995 reported that B-2 microglobulin amyloids (dialysis-related Amyloidosis) precipitated in uraemic patient’s hands in those with long-term hemodialysis, such amyloid deposition may cause carpal tunnel syndrome, trigger finger and joint swellings and erosions. They found that Digital flexor tendon lesions responsible for trigger finger or restriction of active flexion were seen in 21.5% of patients [30]. One case report found that amyloid particles infiltrated flexor tendons and fibrous sheath which was thickened, and carpal tunnel syndrome accompanied TF due to amyloid particles infiltrating the median nerve [6].

TF may occur in familial amyloid polyneuropathy (FAP) (hereditary type) which is systemic Amyloidosis characterized by progressive polyneuropathy, autonomic failure, cardiomyopathy, and connective tissue dysfunction. In a case report, Uotani et al., 2006 found that Tf is the first manifestation of FAP; after that the patient develops carpal tunnel syndrome and cardiomyopathy [31]. TF may occur in age-associated amyloid deposition. Cordiner-Lawrie et al., 2001 found the presence of localized amyloid deposition in the tendon sheath of 11 of 47 TF cases (23%) of idiopathic primary TF (nondiabetic and non-dialysis patients). These amyloid deposits were only found in patients aged over 46 years old and were present around cells and at sites of mucinous and at fibrinoid degeneration which contained highly sulfated glycosaminoglycans [32].

Hypothyroidism is associated with various musculoskeletal diseases since, thyroid hormones play an important role in the development, maturation, and maintaining morphological and functional integrity of the musculoskeletal structures [33]. Cakir et al. 2003 found that TF occurred in 2.9% of patients with thyroid diseases [34].

TF is very common in rheumatoid disease. It is usually caused by nodular tenosynovitis inside the fibrous tendon sheaths of the flexors of the fingers [10]. Helal 1971 found that distal profundus entrapment accounts for 22% of the various pathological sites in rheumatoid patients. He found that this is due to space-occupying synovitis within the flexor sheath distal to the passage of profundus through the tunnel formed by the two heads of insertion of the superficial tendon (Camper’s Chiasm), or sometimes it is due to adhesion between the superficial and deep tendons at this site [35].

In some cases, it was found that snapping doesn’t occur at the palmar aspect of the finger as in typical cases but at the side of the fingers. In these cases, triggering is produced by disturbance of the volar gliding of the lateral bands of the extensor apparatus on the lateral aspects of the proximal interphalangeal joint due to lateral tendon snapping over thickened bony contour or capsular structures [10].

TF is one of the common complications associated with acromegaly. Tagliafico et al.,2009 found that Tf was observed in 25% of the acromegalic patients but none of the control participants. The A1 pulley was significantly thicker in the acromegalic patients but normalized after 1 year in patients who were treated for the disease. In patients with the uncontrolled disease, the condition remained unchanged [15].

Some space-occupying lesions in the tendon bed can constrict the tendon sheath, leading to triggering. Lesions include exostosis, sesamoids, osteochondroma, anomalous insertions of the lumbrical muscles, hematomas within anomalous muscle insertions, tumors of the tendons and tendon sheaths, post-traumatic capsule, ligament and tendon lesions, fractures, cartilage lesions [36].

Turret exostosis, (or acquired osteochondroma) (solitary exostosis), is a dome-shaped, extracortical mass arising from the dorsum of the middle or proximal phalanges of the fingers. It is believed to be triggered by an injury, which eventually leads to areas of mature bone formation. It can be manifested as a subcutaneous nodule [37]. Multiple exostosis disease (also called numerous hereditary osteochondromas or multiple hereditary osteochondromas) is a rare genetic disease with autosomal dominant transmission. There is a family history in about 60% of cases. Exostosis is composed of bone tissue with a peripheral cortex, central spongy bone, and a cartilaginous cap [38]. The common presentation is brachydactyly, phalangeal and metacarpal cone-shaped epiphyses, and clinodactyly. Exostosis or osteochondroma can constrict the tendon sheath, leading to triggering and locking [36].

Sesamoid bones are usually small and ovoid-shaped and can vary in shape and size. Sesamoid bones are found on the palmar and plantar articular surfaces where tendons run near bones and joints. It is thought that they play an essential function as part of the lubricating mechanism, which protects the tendon and decreases friction; however, due to variation in the size, shape, or position of the sesamoid bone itself or anatomical variation in the orientation of the condyles of the phalanx to one another TF can occur [39]. Brown and Ralph, 1992 found that intermittent triggering of the thumb interphalangeal joint was due to sesamoid bone. There was a small amount of inflammation about the sesamoid, which was covered with a thin fibrinous layer [39].

Intramuscular lipoma may arise from the flexor digitorum at the wrist, which leads to triggering and carpal tunnel syndrome [40]. Lipoma can affect the flexor tendon sheath at the fingers leading to TF [41]. The giant cell tumor of the tendon sheath is the second most common soft tissue tumor of the hand and wrist, after the synovial ganglion. Rahimawati et al., 2010 reported a case of giant cell tumor arising from and surrounding the flexor digitorum superficialis and profundus tendons [42].

Leiomyoma is a benign proliferation of smooth muscle mesenchyme. Harb et al., 2009 reported a case of leiomyoma infiltrating the A1 pulley and flexor digitorum superficialis [43]. A tumor mass may accompany flexor tendons at the wrist, causing triggering under the flexor retinaculum (hemangioma, ganglion cyst, schwannoma, or gout tophi [44].

Hyperextension injury to the finger can lead to triggering, it is possibly due to tears in the tendon sheath which may heal with fibrosis and stenosis of the sheath [45]. Penetrating injury on the palm can lead to a tear of the flexor tendon of the finger which leads to finger triggering [46]. Anatomical variation of lumbrical muscle was found in some cases to be the cause of finger triggering. In these cases, lumbrical muscle is inserted in the flexor digitorum superficials to be constricted in the A 1 pulley instead of insertion in the lateral expansion [47]. Envenomation injuries may result in Tf and tenosynovitis. Rattlesnakes, catfish, Stonefish, or stingrays are possible animals implicated in such injuries according to some case reports.

It is thought that this triggering is secondary to an infection, retained micro-foreign bodies, or persistent inflammatory response to the toxin [48].

Dupuytren’s contracture is a pathologic production and deposition of collagens creating nodules and cords in the palm and fingers. It can eventually lead to flexion contracture of joints and severely limit hand function. Some patients with Dupuytren’s contracture can develop TF [49].

Burgess and Watson, 1987 concluded that TF in Dupuytren’s patients could be separated into two categories which are patients with external compression from contracting vertical septa or patients in whom the tendon constriction appeared unrelated to the overlying fascial disease according to intraoperative dealing with these patients [50].

Raynaud’s phenomenon is episodic vasospasm and ischemia of the extremities in response to cold or emotional stimuli, which result in a characteristic color change in extremities (usually fingers) from white, to blue, to red. Raynaud’s phenomenon may be primary or secondary to an underlying condition. In 10–20% of cases, it may be the first presentation of, or may precede the onset of, a connective tissue disease (such as scleroderma or mixed connective tissue disease) [51].

In one case report, a 39-yr-old man who had a history of Raynaud’s phenomenon developed TF for his ten fingers, but after he have changed his occupation to a laborer 3 months before finger triggering [52]. In another case, a 19-yr-old patient with a history of Raynaud’s phenomenon and a diagnosed linear scleroderma developed TF for his ten fingers [52]. Since Raynaud’s phenomenon in secondary type may be connected to connective tissue disease, this may interpret developing TF in such cases.

Since some classes of drugs (fluoroquinolones, glucocorticoids, aromatase inhibitors, and statins) have been suggested to be associated with the risk of 14tendinosis, tenosynovitis, and/or tendon rupture, they can be associated with TF [11].

Special attention was drawn to statins. Eliasson et al, 2019 found that current users of statins had a higher incidence of TF [53]. Since statins are usually prescribed for patients with arterial hypertension, this could explain why arterial hypertension is associated with TF [54]. Since TF is a multifactorial disease, the degree to which occupation is a risk factor may not be easily identified. Sperling,1952 attempted to produce snapping fingers in himself. He flexed his little finger of the right hand at all three joints for periods of 72 hours against the resistance of a powerful spiral spring. A total of about 9,000 flexional movements were made, pure flexion being aimed at. Generalized swelling and tenderness of the flexor side of the finger appeared at once, and a TF developed. He repeated this experiment on other fingers with the same results. He concluded that “numerous small movements, which individually are neither abnormal nor strenuous, may lead to the condition when the effects of these movements are summed up” [55]. This means that highly repeated finger movements may lead to TF. Yavari et al., 2010 presented a case of multiple TFs in a musician; the affected fingers were the actual fingers used for playing the guitar. He had normal plain radiographs. The patient was negative for thyroid function, diabetes, renal disease, gout, and rheumatoid arthritis [56]. Trezise et al., 1998 investigated the occupation histories of 178 TF patients; they concluded that most trigger fingers develop for reasons other than occupation [57].

## Conclusion

A trigger finger is a painful popping or clicking sound which is elicited by flexion and extension of the involved digit. Since various risk factors are associated with TF, clinicians should take history and examine every patient well to define and deal with possible risks. Clinicians should examine the hand for all possible affection sites other than A1 pulley.

## Data Availability

Not applicable.

## Abbreviations

TF: Trigger Finger
CTS: Carpal tunnel syndrome
FDS: The flexor digitorum superficialis
